# Impact of iron deficiency anemia on the function of the immune system in children

**DOI:** 10.1097/MD.0000000000005395

**Published:** 2016-11-28

**Authors:** Tamer Hasan Hassan, Mohamed Ahmed Badr, Nehad Ahmed Karam, Marwa Zkaria, Hosam Fathy El Saadany, Doaa Mohamed Abdel Rahman, Doaa Abdallah Shahbah, Salah Mohamed Al Morshedy, Manar Fathy, Asmaa Mohamed Hosni Esh, Amal Mohamed Selim

**Affiliations:** a Department of Pediatrics; b Department of Clinical Pathology, Zagazig University, Zagazig, Egypt.

**Keywords:** anemia, function, immune, iron

## Abstract

The importance of iron deficiency as a public health problem is based ultimately on the seriousness of its consequences on health. The most extensively investigated consequences of iron deficiency involve work performance and immune function. The significance of the effects on work performance is generally accepted. In contrast, data on the influence of iron deficiency on immune function are often perceived as being confusing and contradictory.

We aimed to evaluate the effect of iron deficiency anemia on humoral, cellular, nonspecific immunity, and also the effect on the cytokines that are the key factors of many immunologic steps.

Forty children with iron deficiency anemia and 20 age and sex-matched healthy children were included. All children were subjected to full medical history, thorough clinical examination, complete blood count, iron indices (serum iron, serum total iron-binding capacity, serum ferritin, and transferrin saturation), immunoglobulin assay (IgA, IgG, and IgM), interleukin (IL)-6 serum level, study of T-lymphocyte subsets, and evaluation of phagocytic function of macrophages and oxidative burst activity of neutrophils.

Patients had significantly lower IgG levels, IL-6, phagocytic activity, and oxidative burst of neutrophils than controls, although there was no significant difference between patients and controls with regard to other immunoglobulins and CD4/CD8 ratio. There was significantly positive correlation between serum iron and IL-6 serum level.

We concluded that humoral, nonspecific immunity (phagocytic activity and oxidative burst), and the IL-6 are influenced in patients with iron deficiency anemia. Study of these abnormalities after correction of iron deficiency is strongly needed.

## 1 Introduction

Iron plays an essential role in immunosurveillance, because of its growth-promoting and differentiation-inducing properties for immune cells and its interference with cell-mediated immune effector pathways and cytokines activities.^[1]^

Iron deficiency anemia is a problem of serious public health significance that impacts mental and physical development, health maintenance, and work performance. It is the most common micronutrient deficiency worldwide. It exceeds 50% in developing countries and is usually attributed to inadequate nutrition.^[2]^

Egypt demographic health survey in 2005 reported a 48.5% prevalence of iron deficiency anemia among Egyptian children.^[3]^

Iron deficiency anemia due to nutritional deficiency is not just a disease of developing countries, but it can also be seen in developed countries. Worldwide, over 40% of children who have iron deficiency anemia are frequently associated with infections.^[4]^

Experimental evidence in the last decades shows that iron is a fundamental element for normal development of the immune system. Its deficiency affects the capacity to have an adequate immune response**.** The role of iron for immunity is necessary for immune cell proliferation, particularly lymphocytes, associated with the generation of a specific response to infection. Humoral immunity appears to be less affected by iron deficiency than is cellular immunity.^[5]^

Iron is required for monocyte/macrophage differentiation, while macrophages require iron as a cofactor for the execution of important antimicrobial effector mechanisms, including the nicotinamide adenine dinucleotide phosphate hydrogen-dependent oxidative burst.^[6]^

Little is known concerning the effect of clinical iron deficiency on cytokines, although it has been reported that in vitro production of cytokines by lymphocytes of iron deficiency patient may be impaired.^[7]^

We aimed not only to evaluate the effect of iron deficiency anemia on humoral, cellular, nonspecific immunity but also the effect on the cytokines that are the key factors of many immunologic steps.

## 2 Methods

This study was conducted in pediatric hematology unit of Zagazig University Hospitals, Egypt, during the period from January 2014 to December 2015. It included 40 children diagnosed to have iron deficiency anemia and 20 age and sex-matched healthy children as a control group.

## 3 Subjects

### 3.1 Patient group

#### 3.1.1 Diagnosis of iron deficiency anemia was based on

1. Hemoglobin (Hb) levels below 2 standard deviations of the age-matched levels (below 11 g/dL, regardless of sex).
2. Serum level of ferritin below 10 ng/mL.
3. Transferrin saturation below 10%.
4. Mean corpuscular volume (MCV) less than 80 femtoliters (FL); mean corpuscular Hb (MCH) less than 27 picograms (pg); iron serum less than 58 μg/dL.

#### 3.1.2 Inclusion criteria

1. All patients should have normal growth and normal serum albumin levels.
2. Nutritional iron deficiency is the only etiology with no other nutritional deficiencies.

#### 3.1.3 Exclusion criteria

1. Previous iron replacement therapy.
2. Cases with a history of taking immunosuppressant drugs, radiotherapy, or chemotherapy. Children with any disease that can affect serum antibodies such as immunodeficiency disease, autoimmune disease, malignancy, and chronic infection.
3. Iron deficiency anemia secondary to non-nutritional causes.
4. Other nutritional deficiencies
5. Patient with impaired growth and low serum albumin levels.

### 3.2 Control group

It included 20 healthy children. Their Hb, hematocrit, and MCV levels should be within normal values of the same age and sex.

All children were subjected to

1. Full medical history and thorough clinical examination;
2. Complete blood count;
3. Iron indices, including serum iron, serum total iron-binding capacity (TIBC), serum ferritin, and transferrin saturation;
4. Assessment of humoral immunity: Immunoglobulin assay was performed using quantitative turbiditmetric test for measurement of IgA, IgG, and IgM in human serum (Spineract, S.A.U., Girona, Spain).
5. Assessment of cytokine production: IL-6 serum level using AviBion Human IL-6 ELISA kit (Ani Biotech Oy; Orgenium Laboratories Business Unit, Vantaa, Finland).
6. Assessment of cellular immunity: Percentage of T-lymphocyte subsets by flowcytometry (FACS-Calibur cytometer; BD Biosciences, CA, San Jose) using antihumanCD4/FITC, antihuman CD8/PE (Dako Multimix TM, Dako Denmark A/S, Denmark).
7. Assessment of innate nonspecific immunity: Phagocytic function of macrophages was evaluated by following the uptake of candida albicans (Sigma, St. Louis, MO) and Intracellular killing test using nitroblue tetrazolium (NBT) test to evaluate neutrophil killing activity (Sigma, St. Louis, MO).

### 3.3 Statistical analysis

The data were checked, entered, and analyzed using SPSS version 11(SPSS Inc., Chicago, IL). Results were expressed as mean ± standard deviation for quantitative variables, and as number and percentage for qualitative ones. Unpaired Student *t*-test, Chi-square test, and Pearson coefficient of correlation (*r*) were used when appropriate. *P* values ≤0.05 qualify as significant results and those ≤0.001 as highly significant results.

### 3.4 Ethics

This study was conducted in accordance with the ethical standards of the Helsinki Declaration of 1964, as revised in 2000,^[8]^ and was approved by the institutional review board of faculty of medicine, Zagazig University. Informed consent was obtained from all study participants and/or their caregivers.

## 4 Results

The mean age of patients was 29.08 ± 32.07 months (range from 6 to144 months). They were 23 males and 17 females. The mean age of controls was 26.65 ± 28.99 months (range from 6 to144 months). They were 10 males and 10 females. Patients and controls were matched as regards age and sex (*P* > 0.05)

Pallor, atrophic glossitis, splenomegaly, and pica were present in 100%, 20%, 7.5%, and 2.5% of patients, respectively, while none of controls had any of these clinical manifestations.

Patients had significantly lower levels of Hb (*P* < 0.001), red blood cells (RBCs) (*P* < 0.05), MCV (*P* < 0.001), and MCH (p < .001) and significantly higher platelet counts (*P* < 0.001) than controls.

Patients had significantly lower serum iron, serum ferritin and transferrin saturation, and significantly higher TIBC than controls (Table 1).

**Table 1:**
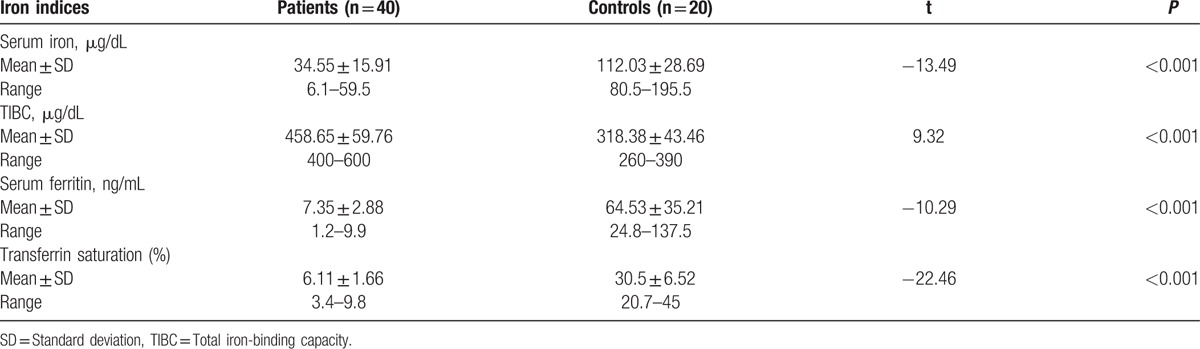
Iron indices in patients and controls.

Patients had significantly lower IgG levels, IL-6, phagocytic activity, and oxidative burst of neutrophils than controls, although there was no significant difference between patients and controls with regard to other immunoglobulins and CD4/CD8 ratio (Table 2).

**Table 2:**
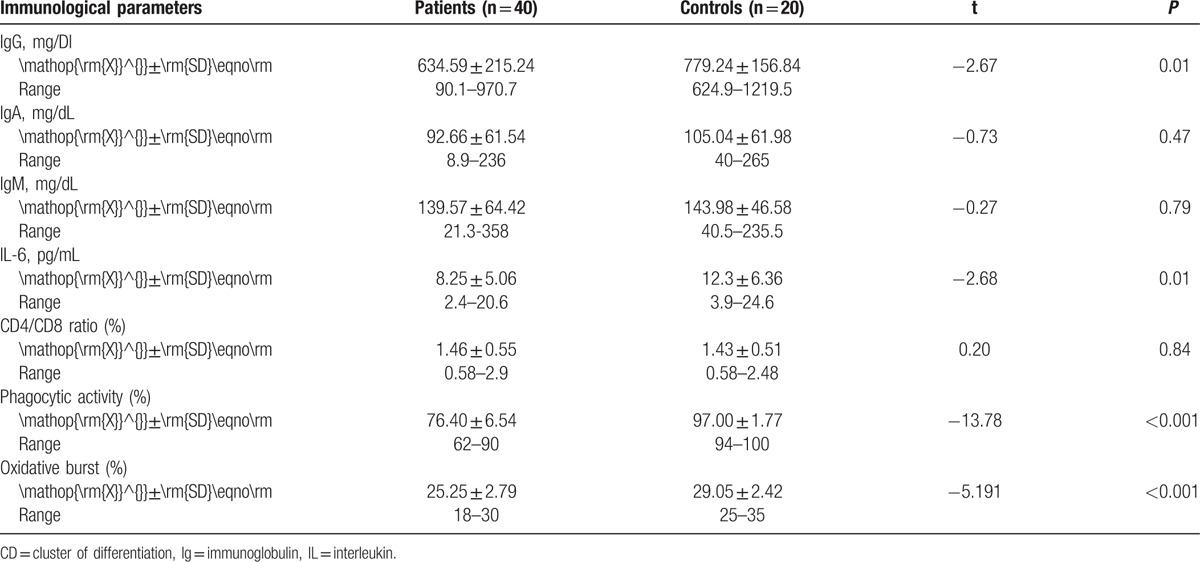
Immunological parameters in patients and controls.

**Figure 1:**
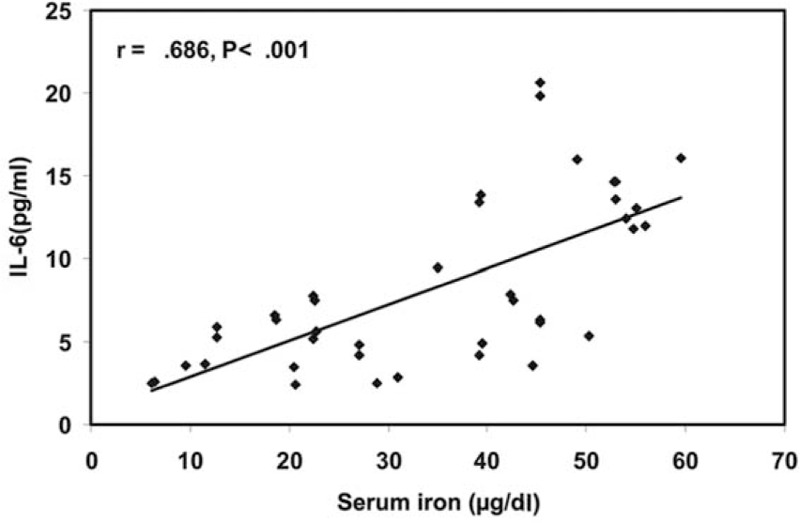
This figure shows that there is significantly positive correlation between serum iron and serum IL-6 level.

There was a significantly positive correlation between serum iron and IL-6, whereas there was no significant correlation between serum iron and other immunological parameters (Fig. 1).

There was no significant correlation between other iron indices (serum ferritin, TIBC, and transferrin saturation) and any of immunological parameters.

## 5 Discussion

Experimental evidence in the last decades shows that iron is a fundamental element for normal development of the immune system. Its deficiency affects the capacity to have an adequate immune response. The role of iron in immunity is necessary for immune cell proliferation.^[9]^

In our study, IgG levels were significantly lower in patients than controls, while there was no significant difference between patients and controls with regard to IgA and IgM levels.

Ekiz et al^[10]^ investigated serum levels of IgG, IgA, IgM, and IgG subgroups IgG1, IgG2, IgG3, and IgG4 in the iron deficiency anemia patients and controls. They found no significant difference except IgG4 levels that were significantly lower in the iron deficiency anemia group (16.7 ± 16.6 mg/dL in children with IDA vs 51.8 ± 40.7 mg/dL in healthy children, *P* < 0.05).

Feng et al^[11]^ found that the mean concentration of serum IgG4 and IgG1, and pneumococcal polysaccharides specific IgG1, IgG2 antibodies were decreased in children with iron deficiency compared with age-matched healthy children.

On the contrary, Bagchi et al,^[12]^ Macdougall et al,^[13]^ and Walter et al^[14]^ had examined antibody-mediated immunity in details and found that Ig levels appeared to be normal in iron-deficient individuals.

In our study, serum IL-6 levels were significantly lower in iron deficiency anemia patients than controls. The more interesting was our observation that there was a significantly positive correlation between serum iron and IL-6, although there was no significant correlation between serum iron and other immunological parameters.

In agreement with our results, Ekiz et al ^[10]^ detected a statistically significant difference between the iron deficiency anemia group and the control group (5.6 ± 3.1 pg/mL in children with IDA vs 10.3 ± 5.3 pg/mL in controls, *P* < .001).

Feng et al ^[11]^ reported decreased levels of IL-6 in patients with iron deficiency anemia and they reported that T-cell dysfunction may be the result of low cytokine activity. It has been shown that removal of iron stores from the body causes a decrease in T-cell proliferation and differentiation with subsequent cytokine secretion.^[7]^

Bergman et al^[15]^ studied in vitro cytokine production in patients with iron deficiency anemia and found that there was no difference in the production of the tested cytokines between patients and controls. The addition of iron to the culture medium did not affect the secretion of interleukin (IL)-2 and IL-1β, but it caused an increase in IL-6, tumor necrosis factor-alpha (TNF-α), and IL-10 production. While Sipahi et al^[16]^ demonstrated no difference in serum levels of IL-6 in iron deficiency anemia before and after iron supplementation.

On the contrary, Jason et al^[7]^ in their study of children with chronic iron deficiency reported decreased level of IL-8 and increased IL-6 level. The level of IL-6 did not change after iron supplementation.

The significant positive correlation that was observed in our study between iron and IL6 can be explained on the basis of the established reciprocal (cause–effect) relationship between iron and cytokine production. Although the secretion of certain cytokines is affected by a deficiency of iron, cytokines are important in maintaining the intracellular iron balance.^[10]^

The exact mechanisms of iron deficiency on the immune system are not yet known, but some authors have suggested that altered levels of some ILs and cytokines (e.g., IL2, IL1, IL6, TNF-alpha, interferon-gamma, IL-4, IL-12p40, and IL-10) might lead to immune system impairments in iron-deficient patients.^[17]^ It has been suggested that altered cell marker expression may contribute to reduced T-cell proliferation during iron deficiency.^[18]^ Iron is essential for enzymes such as ribonucleotidereductase, and it is involved in DNA synthesis; so, the proliferative phase of lymphocyte activation is a Fe-requiring step and it can be diminished during IDA.^[19]^

The identification of hepcidin has enabled a better understanding of the relationship between the immune system and iron homeostasis.^[20]^ Hepcidin, the synthesis of which by the liver is strongly induced by IL-6,^[21]^ inhibits duodenal absorption of iron and blocks iron release from macrophages.^[22]^

In our study, we found no significant change in the T lymphocyte numbers and CD4/CD8 ratio in cases with iron deficiency anemia compared with controls.

Our results are matched with those of Ekiz et al^[10]^ who reported no change in the T lymphocyte numbers and distribution of subgroups in cases with iron deficiency anemia.

On the contrary, Luraschi et al^[23]^ showed a decrease in CD3 and CD8 levels and increase in CD4/CD8 cell ratio. Santos and Falcao ^[24]^ reported a decrease in total lymphocyte numbers and CD3/CD4 cell ratios. Kuvibidila et al ^[25]^ reported decreased T-cell number, blastogenesis, and mitogenesis in response to different mitogens in T lymphocyte in iron deficiency. This alteration is largely correctable with iron repletion. Higgs and Wells ^[26]^ reported impaired cellular immune functions in iron deficiency and its relation to mucocutaneous candidiasis.

In our study, patients had significantly lower phagocytic activity and oxidative burst of neutrophils than controls. Our results are matched with many studies reporting decreased phagocytic and bactericidal activity of neutrophils in iron deficiency anemia.^[14,27,28]^

In their study, Ekiz et al^[10]^ demonstrated a decrease in oxidative burst activity and phagocytic activity in both neutrophils and especially monocytes due to the defects in iron-dependent enzymes wherein the ratio of neutrophils with phagocytic activity was 58.6 ± 23.3% in the anemic group and 74.2 ± 17.7% in the control group (*P* < 0.05). The percentage of neutrophils with oxidative burst activity when stimulated with phorbolmyristate acetate (PMA) was 53.4 ± 32.7% in children with IDA and 81.7 ± 14.3% in the control group (*P* < 0.05).

The NBT dye test was distinctly abnormal in iron-deficient individuals in 2 studies reported by Chandra.^[29,30]^ The abnormality was reversed when the patients were reinvestigated 4 to 7 days after treatment with iron.

Conversely, no abnormality in the NBT dye test was found by either Macdougall et al ^[13]^ or Yetgin et al.^[27]^ However, Yetgin et al^[27]^ did find a highly significant decrease in the magnitude of the oxidative burst (*P* < 0.001) by an assay of hexose monophosphate shunt activity that is more quantitative than the NBT test.

There is strong evidence for a deficit in the ability of neutrophils to kill bacteria. A marked decrease was observed in killing of *Staphylococcus aureus*,^[27]^*Staphylococcus albus*,^[13]^ and *Escherichia coli*.^[27,31]^ Reversal of the abnormality was reported within 4 to 7 days by Chandra ^[30]^ and within <15 days by Walter et al.^[14]^ It was much slower in the study of Yetgin et al ^[27]^ wherein bacterial killing was improved but still very abnormal after 1.5 months of iron therapy and complete after 3 months of therapy.

We concluded that humoral, nonspecific immunity (phagocytic activity and oxidative burst), and IL-6 are influenced in patients with iron deficiency anemia. A study of these abnormalities after correction of iron deficiency is strongly needed.
